# Issues in System Boundary Definition for Substance Flow Analysis: The Case of Nitrogen Cycle Management in Catalonia

**DOI:** 10.1100/tsw.2001.260

**Published:** 2001-10-17

**Authors:** Jordi Bartrola, Maria J. Martin, Miquel Rigola

**Affiliations:** Universitat de Girona, Institut de Medi Ambient, Spain

**Keywords:** substance flow analysis, nitrogen cycle, regional model, system boundaries, scale selection

## Abstract

The great complexity of the nitrogen cycle, including anthropogenic contributions, makes it necessary to carry out local studies, which allow us to identify the specific cause-effect links in a particular society. Models of local societies that are based on methods such as Substance Flow Analysis (SFA), which study and characterise the performance of metabolic exchanges between human society and the environment, are a useful tools for directing local policy towards sustainable management of the nitrogen cycle. In this paper, the selection of geographical boundaries for SFA application is discussed. Data availability and accuracy, and the possibility of linking the results with instructions for decision making, are critical aspects for proper scale selection. The experience obtained in the construction of the model for Catalonia is used to draw attention to the difficulties found in regional studies.

## INTRODUCTION

The nitrogen cycle is a complex matter that has been the subject of many studies and approaches[1,2]. Some nitrogen compounds play a dual role in modern societies: on the one hand they are basic nutrients that in many cases govern agriculture and livestock production, and on the other they are the origin of soil, air, and water pollution problems.

There is a widespread concern in Catalonia (in southwestern Europe), as in many other European regions, about nitrogen pollution, mainly attributed to livestock and agricultural activities. Nevertheless, a comprehensive assessment of the full problem, including other nitrogen pollutant sources like wastewater management, industrial combustion, or transportation, has to be performed to obtain a full overview of the system. In order to tackle nitrogen management, some key questions have to be answered. What are the real problems and their causes? Does any connection among them exist? What are the most efficient preventive strategies for dealing with these situations?

Until now, most pollution abatement and mitigation strategies have been aimed at solving single, specific nitrogen-related pollution problems. Because of the interactions and connections among sets of pollution problems in which reactive nitrogen compounds (ammonia, nitrous oxides, dinitrogen oxide, nitrates) take part, more fully integrated strategies and tactics have to be designed.

The method of Substance Flow Analysis (SFA) is an instrument based on the materials balance principle, which allows a comprehensive description and modelling of the flows and stocks of a single substance, or a group of substances, in the economy and environment of a region[3]. The economy and the environment are divided into several compartments (e.g., industry, combustion process, wastewater management, groundwater) and the flows among them defined and quantified.

A three-step methodology for SFA has been proposed by van der Voet et al.[4], including: (1) definition of the system, (2) quantification of the overview of stocks and flows, and (3) interpretation of the results.

The first step in any substance flow study is to define the system with regard to the aim of the study, the material to be considered, and the space and time to be studied. Generally, two kinds of boundaries have to be set: the *time boundary* and the *space boundary*. The usual time boundary in an SFA study is one year[5,6]. The space boundary may be set at different scales, such as factory, region, nation, river basin, or productive sector. The relevant categories of processes, stocks, and flows belonging to the system must be specified.

The second step in SFA is the quantification of the defined system. This involves identifying and collecting the relevant data and the calculation of the equivalent nitrogen flows.

Finally, two phases can be distinguished in the interpretation of the results: an analysis of sensitivity of the data in order to evaluate the robustness of the overview quantification, and the linking to policy instruments.

The aim of each particular study determines the most appropriate scale or geographical area for the study itself. Foody and Curran[7] recommend considering the scale as a variable that in itself is capable of modifying the manifestation of each of the environmental variables and the interaction among them. Accordingly, the results of studies on nitrogen compounds will also be dependent on the scale, since particular factors and processes may be relevant on one scale but totally absent on another.

The aim of this paper is to discuss the importance of space boundary selection for SFA application at local level and to present the model developed for the selected scale.

## SYSTEM BOUNDARY DEFINITION: MODEL SCALE SELECTION

### Environmental or Administrative System Boundaries

System boundaries can be defined based on environmental or administrative criteria. The selection of the appropriate scale depends on the purpose of the study. In some cases, there are advantages to selecting a scale based on environmental criteria; for instance, for Danube basin water quality management studies[8], as the basin behaves as an ecosystem. If the main interest is to assess the impact of pollution regulation of human activities in a region, the administrative scale will be more convenient.

In our case, there were two main reasons why we chose a system boundary based on administrative criteria:

1. Data availability, with access to the usual collection of statistics based on administrative boundaries.
2. The possibility of linking the results of the study with an environmental policy design.

### Selecting Among Administrative Scales

Studies at different administrative levels are needed. There are examples of material balance[9,10] and substance balance studies[11,12,13] at the national and regional level. The most appropriate administrative scale for a specific study depends on the project objectives and the characteristics of the area under study. Data availability, heterogeneity of the territory, and the potential linkage to policy making are critical aspects to support the selection among administrative scales.

In our case, the potential administrative scales were at the national (Spain), regional (Catalonia), and subregional (county) level.

Since 1986, Spain has been a state member of the European Union. Its territory is divided into 17 autonomous communities and Catalonia is one of them. The territory of Catalonia is administratively separated into four provinces, 41 counties, and 946 municipalities. At the national level, Spanish authorities, like those of other European Union member nations, transpose European Community directives into national legislation and establish minimum standards of environmental quality, while the Catalan government, like that of other autonomous regions inside Spain, has regulatory authority in environmental matters.

The advantages and disadvantages of adopting the different administrative scales for the model are summarised in Table 1 in terms of the availability and quality of the information and the links with policy making.

### *Quantification of the Model*

Almost all the advantages of carrying out the study on a national scale are found in the *quantification of the model* phase (Table1).

On a national scale, there are many official socioeconomic statistics readily available. The opposite situation is found on a county scale, where official statistics are scarce and expert help or specific socioeconomic studies are essential to complete the balance. This lack of information on a county scale is of particular importance for the quantification of imports and exports from system boundaries. The regional scale (Catalonia) would represent an intermediate position. There are official statistics for some important data but there is a lack of information in some aspects, in particular import and export statistics with other regions.

The availability of environmental data shows a different pattern. Flows between processes are more readily available at the regional level because the environmental authority rests with the regional government. The same applies to transboundary input-output flows (e.g., deposition, sea disposal) and information on the technology employed in the processes (e.g., wastewater treatment plants, industrial control technologies), which are better identified at the regional rather than at the national level. Transboundary input-output flows require specific investigation, and this is even easier at a county level. Information about the characteristics of natural systems, such as, for example, the geological properties of soil or aquifers, is rather well known at all scales.

### *Reliability of the Quantification*

The reliability of the quantification for policy making will depend on how representative and accurate the data for the target territory are. When focusing on the interpretation of results as an outcome of the selected scale, the heterogeneity of the territory plays an important role. In most countries, the average values on a national scale may not be representative of the real situation for a fraction of the territory. On a national scale, data accuracy is reduced, as essential information may be unrealistic in some

**TABLE 1**
**Summary of the Advantages of Three Geographical Scales**

| Scale | Spain[a] 500.000 $km^2$ | Catalonia[a] 32.000 $km^2$ | County[a] 143-1733 $km^2$ |
|---|---|---|---|
| **Quantification of the model** | | | |
| (1) Socio-economic statistics: | | | |
| Production and consumption | + + + + | + + + | + |
| Imports and exports | + + + + | + + | + |
| (2) Environmental data | | | |
| Flows between processes | + + | + + + | + + |
| Transboundary input/output flows | + | + + | + + + |
| Characteristics of natural systems | + + + | + + + | + + + |
| Technology employed in processes | + | + + | + + |
| **Reliability of the quantification** | | | |
| Is data representative? | + | + + | + + + + |
| Is data accurate? | + | + + | + + + |
| Emergence of hidden problems | + | + + + | + + + |
| **Linkage to policy-making** | | | |
| Effectiveness of the proposed solutions | + + | + + + | + + + + |
| Jurisdiction on environment issues | + + + | + + + + | + + |
| Financial resources | + + + | + + + + | + + |

a) "+ + + +" Best option, "+" Worse option.

of the aggregates or may be lost through the process of aggregation of heterogeneous data. The final outcome on a national scale is often too simple a picture of the system, which does not allow the establishment of a precise cause-effect relationship. In general, the representativeness and the accuracy that can be achieved at a small scale is greater. At a small scale, the problem lies in the availability and quality of detailed information and data, such as local parameters or specific studies. When applied to smaller territories, standard national values may lack the necessary accuracy and representativeness and induce significant errors.

Finally, the emergence of hidden problems and data verification by cross-checking becomes easier as the scale being considered gets smaller. These considerations about the reliability of the quantification have been taken into account for the quality evaluation of data summarised in Table 1.

### *Linkage to Policy Making*

The aim of the model is to support proposals for effective solutions to environmental issues concerning nitrogen compounds. The effectiveness of the proposed solutions based on model outcomes is higher when the scale is smaller (Table 1), because the specific measures will be more specific and concrete. However, it is not at the county scale where these proposals can best be implemented, because of the limited capacity of environmental policy decision making and the lack of financial resources.

Taking into account the different aspects already explained, the regional scale (Catalonia) is considered the most convenient scale for model development and quantification. There are many positive reasons for the adoption of the regional scale and no negative or restrictive factors against it. Among the positive reasons, the most important are the jurisdictional competence and financial capacity of the Catalan government on environmental matters.

## SUBSTANCE FLOW ANALYSIS OF NITROGEN FOR CATALONIA

SFA methodology has been applied to the study of nitrogen flows in Catalonia with two main objectives. The first objective of the study is to identify the causes and effects of nitrogen flows and the interactions among them. The second objective is the formulation of measures and polices to deal with the detected problems at local level.

### A Description of Catalonia

Catalonia, with an area of 32,113 $km^2$, is located in the northeast of the Iberian Peninsula, on the Mediterranean coast (Fig. 1). The population, over 6 million inhabitants, is unevenly distributed around the territory, with about half of the total population concentrated in the metropolitan area of Barcelona. With an average altitude of 700 meters above sea level, Catalonia combines a wide variety of landscapes and climates, ranging from the wintry, snow-covered Pyrenees (over 2000 meters above sea level) to the Mediterranean-bathed coastline with its pleasant, mild climate.

Tourism, industry, and livestock production are important elements of the Catalan economy. With more than 15 million visitors a year, Catalonia is one of the largest tourist centres on the Mediterranean. The main tourist destinations are concentrated on a continuous stretch of the coastline. Tourism is an important economic activity; it accounts for more than 12% of the Gross Regional Product and employs around 13% of the working population.

Catalonia is the leading industrial region in Spain, contributing 25% of the gross value of Spanish industry. Catalonia's productive structure puts it among the most industrialised regions of Europe. Significant to the nitrogen cycle is the Catalan agro-

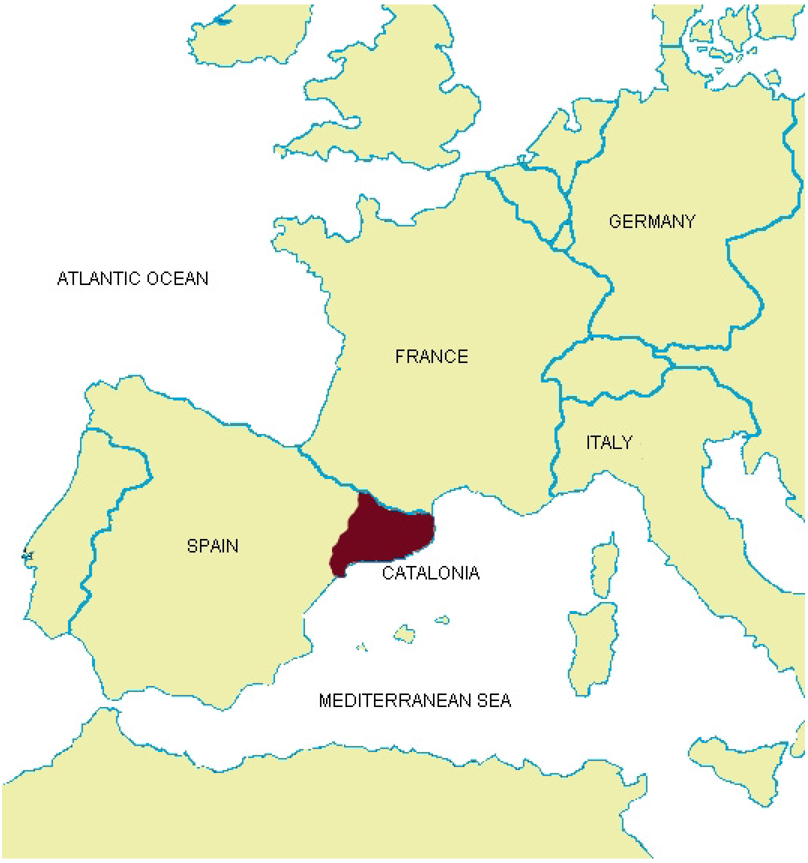

**FIGURE 1.** Geographical location of Catalonia in Europe.

industrial sector, which accounts for more than 22% of the gross value of Spanish agro-industry. The livestock sector, which is characterized by the specialization in livestock fattening, has undergone very strong development and today represents some 60.5% of all agricultural output.

## System Definition

In the system definition of SFA the following processes have been considered for balancing nitrogen compounds (Fig. 2):

| | |
|---|---|
| Agriculture (including soils) | Industry |
| Combustion processes | Livestock |
| Farming | Other soils |
| Groundwater | Private households and the public sector |
| Surface waters | Troposphere |
| Waste management | Wastewater management |
| Water supply | |

The choice of these 13 relevant processes responds to Catalonia's environmental and socioeconomic characteristics. In comparing this study with others[13,14], keep in mind that due to the importance of the livestock production sector in Catalonia, it is taken into account separately. In contrast, forest production is included under *Other soils* process in this figure, due to the relatively low importance of this activity in the Catalan economy. These processes are interconnected by 87 representative fluxes of the nitrogen metabolism in Catalonia. A simplified representation of the system as applied for nitrogen compound balance in Catalonia is shown in Fig. 2. A more detailed description of the interconnections among compartments is depicted in Fig. 3, using as an example the waste water management compartment.

## Issues in Working on a Regional Scale: The Case of Catalonia

The regional scale is not free of particular issues that have to be taken into account during the whole process, from the development of the model to its application in decision making.

With regard to geographic, climatic, and socioeconomic factors, four different subregions can be identified in Catalonia: the coastline, the metropolitan area of Barcelona, the interior region of Catalonia, and the Pyrenees region. Each subregion shows a prevailing source of nitrogen-related environmental problems. The coastline is characterized by the concentration of tourists during summertime, which gives rise to a seasonal effect on demand for goods and products (water, energy, and food) and on the generation of wastes. The metropolitan area of Barcelona, with more than the half of the population of Catalonia, is where

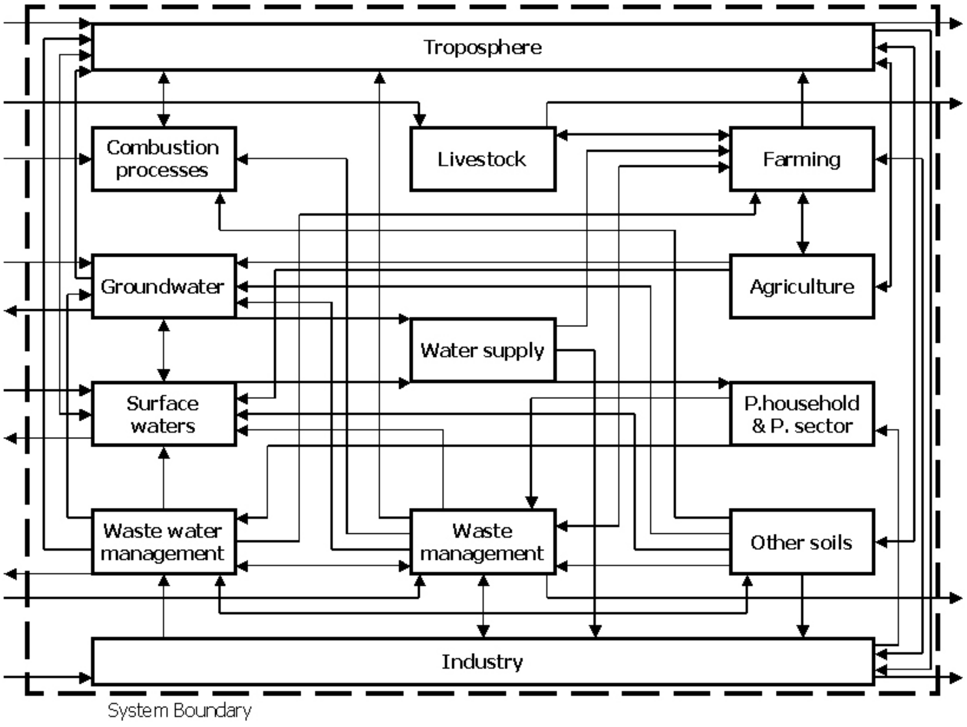

**FIGURE 2.** System definition as applied for nitrogen compound balance in Catalonia. Each box represents a relevant compartment; arrows stand for nitrogen compound flows.

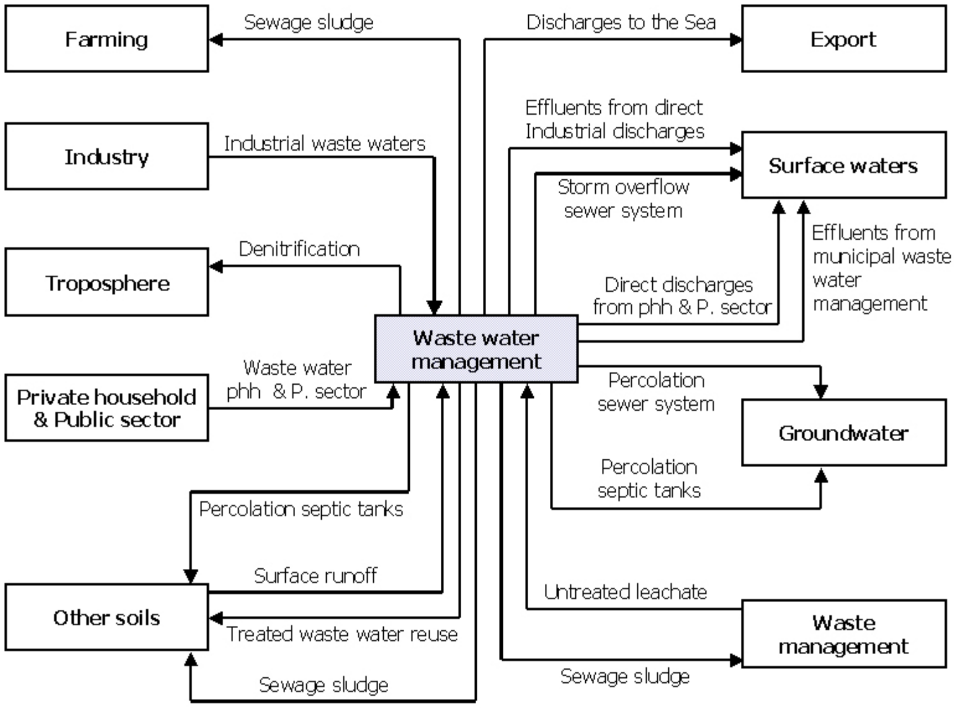

**FIGURE 3.** System definition as applied for nitrogen compound balance in Catalonia considering the wastewater management compartment.

most of the industrial activity is concentrated. The associated nitrogen pollution problems are $NO_x$ emissions from traffic and combustion processes and waste management. Agriculture and livestock production and the corresponding nitrogen pollution problems are mainly found in the interior region of Catalonia. Finally, the Pyrenees area has no significant nitrogen pollution problems.

Another problem is the dispersion of information among many different administrative departments and institutions. Access to this information at the detailed level required for model quantification is not straightforward. Additionally, despite evident progress, delivery of information still lacks transparency from the administration.

## OUTLOOK AND CONCLUSIONS

Pollution by nitrogen compounds is a widespread problem throughout Europe and a significant environmental issue in many specific areas of Catalonia (Spain). For policy development and decision making all of the links between nitrogen pollution and nitrogen flows need to be considered. SFA is a convenient research tool for studying the metabolism of nitrogen compounds throughout the economy and environment of a region and requires the availability of multiple data.

Action is required at local level to solve particular problems, such as the air quality in metropolitan areas or the pollution of specific sources of ground water for human consumption. The complexity of the involved phenomena and the multiplicity of nitrogen compound sources demand a broader approach in the search for cost-effective measures to solve these problems. Planning strategies and decision making must start at a high level, supported by an appropriate regulatory framework and action to implement the decisions. This has been fundamental in developing the SFA model for nitrogen at the Catalan level. Integrating all the causes and effects into such a coherent picture is needed if further progress is to be made in the study of environmental issues concerning nitrogen.

Selection of the model boundaries is primary determined by the objective, although it is influenced by data availability. Any adopted level of scale will have strong and weak points to be taken into account in the definition and the use of the model.

## ACKNOWLEDGMENTS

Mr. Jordi Bartrolí would like to thank the University of Girona for its financial support under grant BR98/946.

| | | |
|---|---|---|
| Received: | July | 19, 2001 |
| Revised: | October | 1, 2001 |
| Accepted: | October | 3, 2001 |
| Published: | October | 17, 2001 |
